# Supplementary material for: Antitubercular activity assessment of fluorinated chalcones, 2-aminopyridine-3-carbonitrile and 2-amino-4H-pyran-3-carbonitrile derivatives: In vitro, molecular docking and in-silico drug likeliness studies
Source: PLoS One. 2022 Jun 16;17(6):e0265068. doi: 10.1371/journal.pone.0265068 (PMC9202851; doi:10.1371/journal.pone.0265068)
Supplement: S1 File — (DOCX) [file pone.0265068.s001.docx]

Antitubercular activity assessment of fluorinated chalcones, 2‐aminopyridine‐3‐carbonitrile and 2‐amino-4H-pyran‐3‐carbonitrile derivatives: *In vitro*, molecular docking and in-silico drug likeliness studies

**Surendra Babu Lagu^1^ ^*^, Rajendra Prasad Yejella^2^,** **Srinath Nissankararao^3^,** **Richie R. Bhandare^4,^ ^5*^, Venu Sampath Golla^2^**, **Bontha Venkata Subrahmanya Lokesh^6^,** **M. Mukhlesur Rahman^7^**, **Afzal Basha Shaik^8*^**

**Supporting Information**

**Table 1.** Antitubercular activity of trifluoromethyl and trifluoromethoxy substituted chalcone derivatives **(1-20).**

| **R"** | **Cmpd #** | | **CLogP** | **H_37_RV**  **MIC (µM)** | **Cmpd #** | **CLogP** | **H_37_RV**  **MIC (µM)** |
| --- | --- | --- | --- | --- | --- | --- | --- |
| 2-chlorophenyl | **1** | 5.03 | | **40** | **11** | 4.84 | 153 |
| 3-chlorophenyl | **2** | 5.03 | | 161 | **12** | 4.85 | 153 |
| 2,3-dichlorophenyl | **3** | 5.49 | | 145 | **13** | 5.29 | 138 |
| 2-nitrophenyl | **4** | 3.77 | | 156 | **14** | 3.62 | 148 |
| 3-nitrophenyl | **5** | 3.84 | | 155 | **15** | 3.65 | 148 |
| 4-nitrophenyl | **6** | 3.86 | | 156 | **16** | 3.15 | 148 |
| 2-thienyl | **7** | 4.38 | | 177 | **17** | 4.19 | 168 |
| 2-fururyl | **8** | 3.72 | | 188 | **18** | 3.55 | 177 |
| 2-pyrrolyl | **9** | 3.52 | | 189 | **19** | 3.39 | 178 |
| 3-indolyl | **10** | 4.50 | | **40** | **20** | 4.32 | **38** |
| - | **Ciprofloxacin** | -0.725 | | 9 | **Pyrazinamide** | -0.676 | 25 |
| - | **Streptomycin** | -3.459 | | 11 |  |  |  |

**Table 2.** Antitubercular activity of trifluoromethyl and trifluoromethoxy substituted 2‐amino-pyridine‐3‐carbonitrile derivatives **(21-40).**

| **R"** | **Cmpd #** | **CLogP** | **H_37_RV**  **MIC (µM)** | **Cmpd #** | **CLogP** | **H_37_RV**  **MIC (µM)** |
| --- | --- | --- | --- | --- | --- | --- |
| 2-chlorophenyl | **21** | 4.90 | 33 | **31** | 4.73 | 32 |
| 3-chlorophenyl | **22** | 4.89 | 134 | **32** | 4.72 | 128 |
| 2,3-dichlorophenyl | **23** | 5.41 | 31 | **33** | 5.25 | 30 |
| 2-nitrophenyl | **24** | 3.61 | 33 | **34** | 3.45 | 31 |
| 3-nitrophenyl | **25** | 3.61 | 130 | **35** | 3.41 | 125 |
| 4-nitrophenyl | **26** | 3.58 | 33 | **36** | 3.44 | 31 |
| 2-thienyl | **27** | 3.83 | **18** | **37** | 4.21 | **17** |
| 2-fururyl | **28** | 3.70 | 38 | **38** | 3.55 | 36 |
| 2-pyrrolyl | **29** | 3.60 | 152 | **39** | 3.42 | 145 |
| 3-indolyl | **30** | 4.49 | **17** | **40** | 4.34 | **8** |
| - | **Ciprofloxacin** | -0.725 | 9 | **Pyrazinamide** | -0.676 | 25 |
| - | **Streptomycin** | -3.459 | 11 |  |  |  |

**Table 3.** Antitubercular activity of trifluoromethyl and trifluoromethoxy substituted 2‐amino-4H-pyran‐3‐carbonitrile derivatives **(41-60).**

| **R"** | **Cmpd #** | **CLogP** | **H_37_RV**  **MIC (µM)** | **Cmpd #** | **CLogP** | **H_37_RV**  **MIC (µM)** |
| --- | --- | --- | --- | --- | --- | --- |
| 2-chlorophenyl | **41** | 4.59 | **17** | **51** | 4.43 | **16** |
| 3-chlorophenyl | **42** | 4.62 | 133 | **52** | 4.44 | 127 |
| 2,3-dichlorophenyl | **43** | 5.11 | 30 | **53** | 4.95 | 29 |
| 2-nitrophenyl | **44** | 3.33 | 32 | **54** | 3.19 | 31 |
| 3-nitrophenyl | **45** | 3.31 | 129 | **55** | 3.16 | 124 |
| 4-nitrophenyl | **46** | 3.32 | 32 | **56** | 3.17 | 31 |
| 2-thienyl | **47** | 4.08 | 36 | **57** | 3.90 | 34 |
| 2-fururyl | **48** | 3.42 | 38 | **58** | 3.27 | 36 |
| 2-pyrrolyl | **49** | 3.29 | 151 | **59** | 2.58 | 144 |
| 3-indolyl | **50** | 4.22 | **16** | **60** | 4.06 | **16** |
| - | **Ciprofloxacin** | -0.725 | 9 | **Pyrazinamide** | -0.676 | 25 |
| - | **Streptomycin** | -3.459 | 11 |  |  |  |

**Table 4.** Binding interactions of trifluoromethyl and trifluoromethoxy substituted chalcone derivatives **(1-20).**

| **R"** | **Cmpd #** | **Binding Energy (kcal/mol)** | **Hydrogen Bonding Chain A** | **Cmpd #** | **Binding Energy (kcal/mol)** | **Hydrogen Bonding Chain A** |
| --- | --- | --- | --- | --- | --- | --- |
| 2-chlorophenyl | **1** | -7.92 | ILE:94, PHE:3, ACA:7, ACA:6, ASP:27, THR:113, THR:46, GLY:15 | **11** | -7.51 | GLY:15, THR:46, ALA:7 |
| 3-chlorophenyl | **2** | -7.32 | LEU:24, LEU:28, TRP:22, THR:46,  GLY:15, MET:20, SER:49, GLU:17 | **12** | -7.25 | SER:49, THR:46, ALA:7, PHE:31,  ILE:5, GLY:15 |
| 2,3-dichlorophenyl | **3** | -8.31 | GLY:15, ILE:94, THR:46, THR:113, ALA:6, TRP: 30, PHE:31 | **13** | -8.04 | TYR:39, ARG:160, ARG:95, ASP:163 |
| 2-nitrophenyl | **4** | -7.76 | THR:46, THR:113, GLY:95, ALA:6 | **14** | -7.59 | THR:46, THR:113, ILE:94, PHE:30,  TRP:30, GLY:15, GLY:96 |
| 3-nitrophenyl | **5** | -8.15 | ILE:50, THR:46, GLY:15, PHE:31,  LEU:28, LYS:32 | **15** | -8.12 | ILE:50, THR:46, GLY:15, PHE:31,  LEU:28, LYS:32 |
| 4-nitrophenyl | **6** | -7.16 | LEU:28, ARG:57, ILE:50, ACA:7, TYR:100, LYS:32 | **16** | -6.98 | ILE:50, LEU:28, LYS:32, ARG:57,  PHE:31, THR:113 |
| 2-thienyl | **7** | -6.92 | ALA:7, GLY:15,THR:46 | **17** | -6.95 | THR:46, ASP:27, ALA:7, PHE:3 |
| 2-fururyl | **8** | -6.76 | ASP:27, GLY:15, THR:46 | **18** | -6.77 | ASP:27, THR:46, SER:49, ALA:7 |
| 2-pyrrolyl | **9** | -6.92 | LEU:24, LEU:28, TRP:22, THR:46,  GLY:15, MET:20, SER:49, GLU:17 | **19** | -6.93 | LEU:24, LEU:28, TRP:22, THR:46,  GLY:15, MET:20, SER:49, GLU:17 |
| 3-indolyl | **10** | -7.95 | TYR:100, ALA:6, ALA:7, THR:46, THR:123, ARG:98 | **20** | -7.81 | TRP:22, GLY:15, THR:46, LEU:24 |
|  | **Isoniazid** | -10.0287 | ARG:52, ASN :182, TRP : 22 |  |  |  |

**Table 5.** Binding interactions of trifluoromethyl and trifluoromethoxy substituted 2‐amino-pyridine‐3‐carbonitrile derivatives **(21-40).**

| **R"** | **Cmpd #** | **Binding Energy (kcal/mol)** | **Hydrogen Bonding Chain A** | **Cmpd #** | **Binding Energy (kcal/mol)** | **Hydrogen Bonding Chain A** |
| --- | --- | --- | --- | --- | --- | --- |
| 2-chlorophenyl | **21** | -8.61 | TYR:100, ALA:7 | **31** | -8.69 | TRP:22, LEU:28, MET:20, TYR:100, ALA:7 |
| 3-chlorophenyl | **22** | -8.11 | ILE:50, THR:46, GLY:15, PHE:31,  LEU:28, LYS:32 | **32** | -7.82 | ILE:50, THR:46, GLY:15, PHE:31,  LEU:28, LYS:32 |
| 2,3-dichlorophenyl | **23** | -9.12 | ALA:7, GLY:15, MET:20, TGR:46, TRP:22, LEU:24, LEU:28 | **33** | **-9.29** | SER:49, HIS:45, GLU:17, THR:46,  THR:123, ARG:98 |
| 2-nitrophenyl | **24** | -8.03 | ALA:7, ALA:19, TYR:100, GLY:96, GLY:15, MET:20, TRP:22 | **34** | -8.28 | GLY:15, LEU:28, ALA:7, TYR:100, MET:20, TRP:22, THR:46 |
| 3-nitrophenyl | **25** | **-9.33** | LEU:24, LEU:28, TRP:22, THR:46,  GLY:15, MET:20, SER:49, GLU:17 | **35** | -9.05 | LEU:24, LEU:28, TRP:22, THR:46,  GLY:15, MET:20, SER:49, GLU:17 |
| 4-nitrophenyl | **26** | -7.97 | SER:49, ILE:50, TYR:100, ARG:52, LYS:32 | **36** | -7.94 | PHE:31, LEU:28, LYS:32, ARG:52,  TYR:100, SER:49 |
| 2-thienyl | **27** | -7.85 | LEU:24, ALA:7, TYR:100, GLY:96, GLY:15, TRP:22 | **37** | -8.06 | SER:49, GLU:17, HIS:45, HIS:46,  MET:16, ARG:96, THR:123, GLY:15 |
| 2-fururyl | **28** | -7.72 | SER:49, HIS:45, GLU:17, GLY:15, ILE:94, TYR:100 | **38** | -7.6 | HIS:45, SER:49, THR:46, MET:16,  ARG:96, TYR:100 |
| 2-pyrrolyl | **29** | -7.85 | ILE:50, THR:46, GLY:15, PHE:31,  LEU:28, LYS:32 | **39** | -8.09 | ILE:50, THR:46, GLY:15, PHE:31,  LEU:28, LYS:32 |
| 3-indolyl | **30** | **-9.15** | ALA:7, TRP:22, LEU:24, ASN:18 | **40** | **-9.67** | ASP:163 [3.0], TYR:39 [2.3], GLU:166 [2.1] |
|  | **Isoniazid** | -10.0287 | ARG:52, ASN :182, TRP : 22 |  |  |  |

**Table 6.** Binding interactions of trifluoromethyl and trifluoromethoxy substituted 2‐amino-4H-pyran‐3‐carbonitrile derivatives (**41-60**).

| **R"** | **Cmpd #** | **Binding Energy (kcal/mol)** | **Hydrogen Bonding Chain A** | **Cmpd #** | **Binding Energy (kcal/mol)** | **Hydrogen Bonding Chain A** |
| --- | --- | --- | --- | --- | --- | --- |
| 2-chlorophenyl | **41** | -9.02 | THR:46, GLY:15, SER:49 | **51** | -8.35 | TYR:100, ALA:7, PHE:31, TRP:22,  LEU:28 |
| 3-chlorophenyl | **42** | -7.73 | LEU:24, LEU:28, TRP:22, THR:46, GLY:15, MET:20, SER:49, GLU:17 | **52** | -7.8 | LEU:24, LEU:28, TRP:22, THR:46,  GLY:15, MET:20, SER:49, GLU:17 |
| 2,3-dichlorophenyl | **43** | **-9.47** | GLY:15, GLU:17, SER:49 | **53** | **-9.87** | GLU:17, SER:49, THR:46, THR:123, ARG:98 |
| 2-nitrophenyl | **44** | -7.57 | THR:46, LEU:24, LEU:28, TRP:22, MET:20, SER:49 | **54** | -7.29 | LEU:24, LEU:28, TRP:22, THR:46,  GLY:15, MET:20, SER:49, GLU:17 |
| 3-nitrophenyl | **45** | -9.37 | ILE:50, THR:46, GLY:15, PHE:31, LEU:28, LYS:32 | **55** | -8.94 | ILE:50, THR:46, GLY:15, PHE:31,  LEU:28, LYS:32 |
| 4-nitrophenyl | **46** | -7.76 | THR:46, MET:16, GLU:17, SER:49, PHE:31, LEU:28 | **56** | -7.3 | ILE:50, THR:46, GLY:15, PHE:31,  LEU:28, LYS:32 |
| 2-thienyl | **47** | -7.78 | GLY:96, THR:46, TYR:100, LEU:28 | **57** | -8.27 | ARG:98, GLY:15, ALA:7, MET:16,  THR:46, THR:123 |
| 2-fururyl | **48** | -8.17 | THR:46, ARG:98, GLU:17, SER:49 | **58** | -7.95 | ALA:7, GLY:95, GLY:15, THR:46,  THR:123, SER:49, GLU:17 |
| 2-pyrrolyl | **49** | **-9.43** | LEU:24, LEU:28, TRP:22, THR:46, GLY:15, MET:20, SER:49, GLU:17 | **59** | **-9.26** | LEU:24, LEU:28, TRP:22, THR:46,  GLY:15, MET:20, SER:49, GLU:17 |
| 3-indolyl | **50** | **-9.82** | GLU:17, GLY:15, TYR:100, ARG:98, THR:46, SER:49 | **60** | **-9.49** | ALA:7, TRP:22, LEU:28, SER:49,  MET:20, GLY:15 |
| - | **Methotrexate** | -10.0287 | ARG:52, ASN :182, TRP : 22 |  |  |  |

**Table 7.** Other interactions of compound **40** against 1G3U protein.

| **Compound ID** | **PROTEIN CHAIN-A** |
| --- | --- |
| **40** | ARG:14 [3.3] , ARG:95 [3.1], ARG:74 [3.4], ARG:160 [3.0], ASP:94 [3..1], ASP:70 [3.5], ASP:9 [3.3], PHE:36 [3.3], PRO:37[1.3], SER:99 [2.5], ASN:100 [3.1], TYR:103 [3.3], TYR:165 [3.4], LEU:52 [3.3], MG:300 [3.3], ALA:35 [3.3] |

**Table 8.** Cytotoxicity of 40 against human normal liver cells (IC_50_, µg/mL)^a^.

| **S. No** | **Compound** | **Human normal liver cells (L02)** |
| --- | --- | --- |
| 1 | **40** | >70 |

^a^Mean value from three experiments

**Table 9.** Computed properties using SWISSADME.

| **Compound #** | **GI**  **absorption** | **PgP**  **substrate** | **Lipinski #violations** | **CYP2D6 inhibitor** | **CYP2C19 inhibitor** |
| --- | --- | --- | --- | --- | --- |
| **20** | High | Yes | 0 | Yes | No |
| **37** | Low | No | 0 | No | Yes |
| **40** | High | No | 0 | No | Yes |
| **60** | High | Yes | 0 | No | Yes |
| **Ciprofloxacin** | High | Yes | 0 | No | No |
| **Streptomycin** | Low | Yes | 3 | No | No |
| **Pyrazinamide** | High | No | 0 | No | No |
