# Supplementary material for: Antitubercular activity assessment of fluorinated chalcones, 2-aminopyridine-3-carbonitrile and 2-amino-4H-pyran-3-carbonitrile derivatives: In vitro, molecular docking and in-silico drug likeliness studies
Source: PLoS One. 2022 Jun 16;17(6):e0265068. doi: 10.1371/journal.pone.0265068 (PMC9202851; doi:10.1371/journal.pone.0265068)
Supplement: Attachment — Submitted filename: Rebuttal Letter.docx [file pone.0265068.s002.docx]

Rebuttal Letter

We are very thankful to the academic editor and the esteemed reviewers for their valuable comments. Below are our point-by-point replies for the comments provided on the manuscript.

Additional Editor Comments (if provided):

The manuscript has been reviewed by the eminent reviewers. As you could see that they are advising to do more studies. Keeping in view the recommendations of the reviewers, we are unable to accept the article in the present form. The authors may re-submit after completing the required assays.

**Reply: We have addressed some of the issues raised by the reviewers except for the additional experiments suggested. We are currently facing challenges in funding to perform enzymatic assays and this point will be considered for our future studies.**

Reviewers' comments

Reviewer's Responses to Questions

**Comments to the Author**

1. Is the manuscript technically sound, and do the data support the conclusions?

The manuscript must describe a technically sound piece of scientific research with data that supports the conclusions. Experiments must have been conducted rigorously, with appropriate controls, replication, and sample sizes. The conclusions must be drawn appropriately based on the data presented.

Reviewer #1: No

Reviewer #2: Yes

Reviewer #3: No

2. Has the statistical analysis been performed appropriately and rigorously?

Reviewer #1: N/A

Reviewer #2: N/A

Reviewer #3: No

3. Have the authors made all data underlying the findings in their manuscript fully available?

The [PLOS Data policy](http://www.plosone.org/static/policies.action#sharing) requires authors to make all data underlying the findings described in their manuscript fully available without restriction, with rare exception (please refer to the Data Availability Statement in the manuscript PDF file). The data should be provided as part of the manuscript or its supporting information, or deposited to a public repository. For example, in addition to summary statistics, the data points behind means, medians and variance measures should be available. If there are restrictions on publicly sharing data—e.g. participant privacy or use of data from a third party—those must be specified.

Reviewer #1: Yes

Reviewer #2: Yes

Reviewer #3: Yes

4. Is the manuscript presented in an intelligible fashion and written in standard English?

PLOS ONE does not copyedit accepted manuscripts, so the language in submitted articles must be clear, correct, and unambiguous. Any typographical or grammatical errors should be corrected at revision, so please note any specific errors here.

Reviewer #1: No

Reviewer #2: Yes

Reviewer #3: No

5. Review Comments to the Author

Please use the space provided to explain your answers to the questions above. You may also include additional comments for the author, including concerns about dual publication, research ethics, or publication ethics. (Please upload your review as an attachment if it exceeds 20,000 characters)

Reviewer #1: The paper highlights the antitubercular activity of a variety of fluorinated chalcones, pyridine carbonitriles and their associated pyran derivatives. Moreover, the paper describes the structure activity relationship studies of these molecules and their differing activity against Mycobacterium tuberculosis with an in-silico model to aid the interpretation.

Consequently, this paper is of interest due to its importance to the anti-tuberculosis community however, its striking similarity to the authors full papers highlighted (reference 48 and 49) make it difficult to review in its current form. The data and the discussion in the previous papers describe the synthesis of these molecules, their inception and provide a broader range of activity against bacteria and fungus than that presented here.

1. Notwithstanding the duplicitous nature of the paper, other concerns about the article exist with the first being the introduction that whilst this provides some background to the disease and treatments it would benefit from further detail.

**Reply: Introduction has been exhaustively covered based on the literature survey and we have added Figure 1 covering the drugs used in tuberculosis having heterocyclic ring structures to enhance the relevance and readership of the work.**

2. In the same way, the materials and methods are not sufficiently described to be able to reproduce the work with the synthesis of the molecules omitted and described at the beginning of the results section. This is unnecessary as they have been published before in the two previous papers.

**Reply: The chemistry part in results and discussions has been omitted as per the reviewer’s suggestion.**

3. Nevertheless, the results from the MABA assay are well described and presented in a logical fashion which is easy to understand for the reader, however, the same compounds that are active against Mtb are also active against other gram positive, gram negative and fungi leading to the conclusion that these molecules are non-specific cytotoxic molecules, a characteristic long known for chalcones (Zhou, B.; Xing, C. Diverse molecular targets for chalcones with varied bioactivities. Med. Chem. 2015, 5, 388–404) and perhaps the other architectures as well.
Following this through, leads to the docking studies which given the nature of the molecules and the significant differences between actinobacteria and other gram positive and gram-negative bacteria conclusion that these molecules will act on the same target as others similar in structure is not rigorous. To determine the target of the molecules, mutant bacteria should have been grown, harvested, sequenced and the mutated genes analysed, and the transcribed biological entity subjected to a battery of biochemical investigations to confirm the target. As far as I am aware this study hasn’t been conducted?

**Reply: While we agree with the valuable suggestion given by the reviewer, it is currently challenging to conduct additional experimentation as we have already exhausted the funding sources. We will consider this point as a part of our future study.**

Finally, whilst the conclusion does draw the work together, it cannot be appropriate based on the data and therefore, for all these reasons I recommend rejection of this paper at this time.

…………………………………………………………………………………………………..

Reviewer #2: This manuscript reported the antitubercular activity in-vitro and in-silico molecular modelling for 3 series of derivatives including chalcones, pyridine‐ 3‐ carbonitrile, 4H‐ pyran‐ 3‐ carbonitrile scaffolds. Recently, these series already evaluated by authors for antibacterial activity in two different journals.
The manuscript is well written, and the results are well designed and support the aim of the study. There are no major critical comments and the manuscript deserved publication following some minor corrections.
Minor comments:
1. In the introduction: should be discussing the antibacterial activity for these series that previously published by the authors.

**Reply: The previously published antimicrobial study has been included in the introduction.**

2. In the discussion: should be comparing the antitubercular activity with previously reported the antibacterial and antifungal activity for the same series.

**Reply: As the major focus of the manuscript is on antitubercular activity we don’t feel to discuss the other activities in this study. However, we compared the and discussed the antitubercular activity among fluorinated chalcones (1-20), 2‐amino-pyridine‐3‐carbonitrile (21-40) and 2‐amino-4H-pyran‐3‐carbonitrile (41-60) derivatives.**

3. Include clog P for the compounds in the tables (1-3)

**Reply: clog p for the compounds in tables 1-3 have been included.**

4. In silico drug likeliness studies: for logical comparison, add also the ADME properties for the known antitubercular compound as ciprofloxacin and the other potent compounds from each series in table 7.

**Reply: In silico drug-likeliness studies have been added in table 7 covering known antitubercular compounds and other potent compounds in each series.**

**…………………………………………………………………………………………………..**

Reviewer #3:

1) authors should perform inhibitory assays of compounds against thymidylate kinase enzyme.

**Reply:** We are currently facing challenges in funding to perform enzymatic assays and this point will be considered for our future studies.

2) only docking studies of the compounds cannot determine the right biological target of these compounds.

**Reply: We agree with the reviewer in this regard and enzyme inhibitory assays can further strengthen the results obtained from the docking studies.**

3) Different binding interaction of compounds are showing that they have different modes of action.

**Reply: The two series are chemically different. Hence, they have different conformations and orientations within the binding pocket which enabled the compounds to show different binding interactions.**
