# Supplementary material for: Antitubercular activity assessment of fluorinated chalcones, 2-aminopyridine-3-carbonitrile and 2-amino-4H-pyran-3-carbonitrile derivatives: In vitro, molecular docking and in-silico drug likeliness studies
Source: PLoS One. 2022 Jun 16;17(6):e0265068. doi: 10.1371/journal.pone.0265068 (PMC9202851; doi:10.1371/journal.pone.0265068)
Supplement: Attachment — Submitted filename: Response to reviewers.docx [file pone.0265068.s003.docx]

Rebuttal Letter

We are very much thankful to the academic editor, esteemed reviewers and the office editorial staff for their valuable comments. Below are our point-by-point replies for the comments provided on the manuscript.

Journal Requirements:

Q. We suggest you thoroughly copyedit your manuscript for language usage, spelling, and grammar.

Reply: The manuscript has been thoroughly checked for spelling and grammatical errors and the same has been highlighted in red in the revised manuscript with track changes.

Q. Please review your reference list to ensure that it is complete and correct. If you have cited papers that have been retracted, please include the rationale for doing so in the manuscript text, or remove these references and replace them with relevant current references. Any changes to the reference list should be mentioned in the rebuttal letter that accompanies your revised manuscript. If you need to cite a retracted article, indicate the article’s retracted status in the References list and also include a citation and full reference for the retraction notice.

Reply: All the references have been checked for their correctness and we have found none of the cited papers have been retracted.

Additional Editor Comments (if provided):

Authors have done good work. Although the synthetic part has already been published, the antitubercular activity was carried out. The work could have been substantiated with enzymatic studies. But in my opinion this much work may be published.

Review Comments to the Author

Please use the space provided to explain your answers to the questions above. You may also include additional comments for the author, including concerns about dual publication, research ethics, or publication ethics. (Please upload your review as an attachment if it exceeds 20,000 characters)

Reviewer #1: The authors have presented antitubercular actvities for the previously reported compounds.

1. Introduction section mentions about the WHO date. Please cite the same in references.

Reply: The reference pertaining to section about WHO date has already been previously mentioned in the introduction and has been cited in the reference. The reference number is 1.

2. Docking images are not legible. Especially, the compouds are not legible in the docking images.

Reply: High-quality images of docking are inserted for the compounds 26 and 40 in figures 4 and 5 respectively.

3. Did the authors perform acute cytotoxicity and in vivo analysis?

Reply: Acute animal or in vivo cytotoxicity was not performed. Rather, the most potent compound 40, has been evaluated for in vitro cytotoxicity. The point highlighted by the reviewer is encouraging and is under consideration for our future study.

4. The authors must discuss the previously performed activity of the test compounds that encouraged the appraisal of their antitubercular activity in the present report.

Reply: The suggestion mentioned by the reviewer has been implemented and the following had been added in the introduction part.

Motivated by the excellent antimicrobial activities, we intended to test the target compounds against Mycobacterium tuberculosis in order to identify potential lead molecules against tuberculosis infections. Hence, herein we report the antitubercular evaluation of fluorinated chalcones and their 2‐amino-pyridine‐3‐carbonitrile and 2‐amino-4H-pyran‐3‐carbonitrile derivatives by standard computational and biological methods.

5. Is the clogP evaluated experimentally or theoretically? Please mention the percentage error in either case.

Reply: The clogP value has been retrieved from SwissADME software using smile strings of the target compounds. As it is predicted value employing a online algorithm, incorporating the percentage error is not possible.

6. Since molecular docking forms the only basis of supporting studies, it should be discussed in details for atleast the most active compounds and corroborated with the SAR analysis.

Reply: The molecular docking of the most active compound 40 has been elaborated and correlated with the SAR derived from the in vitro activity data and mentioned in the results and discussions of molecular docking part.
