# Supplementary material for: Antitubercular activity assessment of fluorinated chalcones, 2-aminopyridine-3-carbonitrile and 2-amino-4H-pyran-3-carbonitrile derivatives: In vitro, molecular docking and in-silico drug likeliness studies
Source: PLoS One. 2022 Jun 16;17(6):e0265068. doi: 10.1371/journal.pone.0265068 (PMC9202851; doi:10.1371/journal.pone.0265068)
Supplement: Attachment — Submitted filename: Response to reviewers.docx [file pone.0265068.s004.docx]

Rebuttal Letter

We are very much thankful to the academic editor, esteemed reviewers and the office editorial staff for their valuable comments. Below are our point-by-point replies for the comments provided on the manuscript.

Reviewer #1: The author have presented the anti-tubercular potency of fluorinated chalcones, 2‐aminopyridine‐3‐carbonitrile and 2‐amino-4H-pyran‐3‐carbonitrile derivatives. The molecular docking and in vitro studies support the biological activity of these molecules. The authors must address the following points to improve the manuscript.

1] The introduction section has many claims regarding the morbidity and mortality rate of TB without a valid reference. Even if the WHO statistics are cited, URL must be provided so that the readers benefit form it.

Ans: The URL has been provided in the text.

2] If the synthesis protocol is novel, the percentage yield of the compounds must be reported.

Ans: In “Results and Discussions” under “Chemistry”, a statement “The synthesis and characterization of compounds 1-60 has been published previously [48-49]” has been mentioned in the original manuscript.

3] Drug likeliness is calculated by an online tool. Is there an experimental validation to the reported clogP values?

Ans: The clogP value has been retrieved from SwissADME software using smile strings of the target compounds and the experimental validation has not been done.

4] Is the reported online tool for calculating clogP a valid tool for these studies? What is the incidence of error?

Ans: Yes, the SWISSADME online software is a routinely used tool by medicinal chemists for in silico prediction of physicochemical and pharmacokinetic properties. There are many review articles in medicinal chemistry that highlights the significance and validity of this tool [https://doi.org/10.2174/0929867327666201020151804; <https://doi.org/10.1038/srep42717>; https://doi.org/10.3390/ijms20184612]. As it is predicted value employing online algorithm, incorporating the percentage error is not possible.

5] Which docking tool is used for the studies? The docking data only shows hydrogen bonding interactions. Can the other interactions of molecules with peptide be rejected?

Ans: The molecular docking was performed using AUTODOCK 4.2 version. Other than hydrogen bonding, the compounds also had hydrophobic and halogen bonding interactions which was clearly discussed under “Molecular docking studies” in “Results and Discussions”.

Reviewer #2: Authors have fulfilled all the queries/comments that was asked reviewers previously. Hence, now the manuscript is well written. I believe that it is a nice piece of work for being published in the PLOS ONE. Finally, I recommend that the paper should be accepted for the publication in the present form with some justification or corrections.

1] Mention the full name NIH and PZA in the text?

Ans: The full names of INH and PZA are mentioned in the manuscript.

2] The author mentions in the text that, the docking conformation of compound 40 “showed” good interactions and so on. This is the modelling study, thus “suggests” is the right verb to be used.

Ans: The right verb “suggests” has been used in the manuscript.

3] In docking study authors are also required to mention in what range bond distance of hydrophobic and hydrogen interactions are determined.

Ans: The bond distance for hydrogen and hydrophobic interactions are added in Tables 5 and 7 for compound 40.

4] In conclusion author mentions compound 27 for the docking study while they performed docking with 26 and 40.

Ans: The study indicated that compound 40 was the most potent among others, hence we have removed the docking images for other compounds.

5] The authors need to give some more methological data regarding their analyses as how big or small was the box that the authors used for the molecular docking? Did that cube cover the whole protein or only the binding site? These information need to be provide in methodology.

Ans: The cube covers on the active site i.e. active binding site, and this information has been provided already in the manuscript.
